# A critical review of obstetric and gynecological physical examination videos available on YouTube: Content analysis and user engagement evaluation

**DOI:** 10.1097/MD.0000000000016459

**Published:** 2019-07-26

**Authors:** Hamza Mohammad Abdulghani, Shafiul Haque, Tauseef Ahmad, Mohammad Irshad, Kamran Sattar, Mohammed Meteb Al-harbi, Nehal Khamis

**Affiliations:** a Department of Medical Education; b Research and Scientific Studies Unit, College of Nursing and Allied Health Sciences, Jazan University, Jazan; c Medical Division, Dasman Diabetes Institute, Dasman, Kuwait; d Department of Family and Community Medicine, College of Medicine, King Khalid University Hospital, King Saud University, Riyadh, Saudi Arabia; e Pathology and Medical Education Departments, College of Medicine, Suez Canal University, Ismailia, Egypt.

**Keywords:** clinical examination, obstetrics and gynecology, physical examination, YouTube

## Abstract

**Background:**

Video-sharing website “YouTube” is a growing source of healthcare information. But, the videos uploaded on this open platform are not peer reviewed, therefore, the information available needs to be sufficiently evaluated. No studies have been conducted to evaluate the authenticity and utility of obstetrics and gynecology (Obs/Gyne) physical examination YouTube videos. This study was performed to analyze the sources, contents, and quality of videos about the Obs/Gyne clinical examination available on YouTube.

**Methods:**

A systematic search was performed on YouTube website using the following key words: “OBSTETRIC,” “GYNECOLOGICAL,” “SPECULUM OBSTETRIC,” “OBSTETRIC CLINICAL,” “BIMANUAL PELVIC,” and “EXAMINATION” to analyze the sources, contents, and the quality of YouTube videos about the Obs/Gyne clinical examination during the period between November 2015 and March 2017. The videos were classified into educationally useful and useless based on the content, accuracy of the knowledge, and the demonstration.

**Results:**

Out of total 457 screened videos, 176 (38.51%) videos met the pre-set inclusion criteria. After review, out of 176 pertinent videos, 84 (47.7%) videos were found educationally useful, and out of these 84 useful videos, only 29 (34.5%) were highly educational in nature.

**Conclusion:**

YouTube videos showed variable educational value. Only, a small number of videos were identified as useful and can be used by the medical students for self-directed learning and by the clinical teachers for educational purposes or other academic activities.

**Key Message**

- This is the very first study evaluating the usefulness, authenticity, and preciseness of YouTube's Obs/Gyne physical examination videos.
- The YouTube Obs/Gyne clinical examination videos were classified into educationally useful and useless based on the content, accuracy of the knowledge, and the instructions.
- This study gives an overview about the YouTube videos of Obs/Gyne clinical examination and warrants for proper evaluation of the videos before their use for learning and other academic purposes.

## 1 Introduction

Video watching and sharing websites have influenced the way of teaching and learning in medical science education and health care services training. Advancements in the information and communication technologies have resulted in the enhanced attention of the educational community to multimedia learning resources.^[1]^ The utilization of multimedia videos for education has reformed the way that visual materials were found to be helpful for learners. Therefore, learners are capable of organizing and integrating the information with benefits from these visual materials more efficaciously.^[2,3]^ Moreover, the dynamic visualizations are considered informative learning tools because of their ability to represent the contents in a very simple manner that are difficult to verbalize but easy to demonstrate.^[4]^ Additionally, the multimedia videos present the learners with content and context, along with language, which have greater impact and role in classroom education and self-study.^[5]^ Worldwide, it is evident that the use of multimedia in the form of videos has also increased the percentage of online learners as well as the implementation of evidence-based practice.^[6,7]^

Nowadays, internet-based technologies have become an essential and integral part of the medical students’ life as they provide support towards new emerging learning challenges.^[8,9]^ Users access multimedia educational videos for many purposes, including learning, training, and practice. Medical professionals, medicine students, and patients use mobiles, laptops, and notepads to access the contents of these informative internet videos, which are controlled by different internet video sharing websites.^[10]^

YouTube is one of the most popular and freely accessible video broadcast platforms, having popular sources of information, with more than 100 million viewers daily.^[7,11]^ For medical students, YouTube not only provides access to educational information with a new trend of learning the subject using videos, but it also provides a platform for sharing interactive information, lectures, and other educational material. At present, YouTube has over 100 million videos and has become an important tool of significant financial value, especially for finding videos and holding personal accounts about health and illnesses.^[12]^ Additionally, its power of becoming the most commonly known personalized health education and health communication resource cannot be undervalued.^[13]^ Therefore, in terms of its popularity and convenience, YouTube is viewed as a significant platform for the distribution of relevant healthcare information among medical students. However, there is a risk of dissemination of misleading information as most of the videos are not peer reviewed and/or generally not uploaded by any educational institute or college.^[10]^ Keeping its utility and application in view of our day to day learning, YouTube videos have been evaluated in a number of areas related to medical science education, patient health information, and medical skills.^[13–15]^ As such, there is currently no proof that all the videos available on YouTube are useful in terms of providing educationally useful material. Thousands of videos presented on YouTube encourage dissemination of misleading or distorted information which could possibly be disadvantageous and might endanger some viewers’ practices.^[15]^

The Obs/Gyne physical examination is an important competency required for all medical students. However, a small number of female patients armor the preference for the physical exam by medical students and trainees. In such a situation, these medical students and trainees are bound to rely and to use alternative educational resources. Currently, the use of simulation-based training prior to actual patient exposure/contact is widely used and watching relevant educational videos prior to the simulation based practice and the review and maintenance of these skills is an extensively accepted educational approach.^[11]^

Any types of internet videos are of varying quality, even if we consider the same topic/title. We therefore hypothesized that major differences exist in the quality of these educational internet videos with no significant research work having been done to evaluate the quality or measure the differences in the quality of these internet educational videos. Thus, the aim of this study was to evaluate the usefulness and examine the accuracy of obstetric and gynecological (Obs/Gyne) physical and clinical examination videos available on the YouTube platform. To the best of our information, this is the very first study evaluating the usefulness, authenticity, and preciseness of YouTube's Obs/Gyne physical examination videos.

## 2 Methods

### 2.1 Identification and eligibility of the relevant YouTube videos

A systematic search of internet videos was performed on YouTube website during the period between November 11, 2015 and March 15, 2017. The keywords used during YouTube web searches were: “OBSTETRIC,” “GYNECOLOGICAL,” “SPECULUM OBSTETRIC,” “OBSTETRIC CLINICAL,” “BIMANUAL PELVIC,” and “EXAMINATION.”

### 2.2 Data collection and quality assessment of YouTube videos

The methodological quality assessment of the YouTube videos was performed according to two previously published evaluation criteria with suitable modifications.^[14,15]^ During the YouTube web search only the first 5 web-pages of results, retrieved against each search keyword, were screened for pertinent videos. Over 5 pages of YouTube search results, the results mostly do not match/relate very well to the given query search keyword(s).^[14,15]^ Hence the first 5 web pages were scanned for video retrieval and data collection.

For each retrieved video, the methodological quality assessment and data extraction were independently summarized in duplicate by 2 independent investigators (HMA and TA) using a standard procedure. Two subject expert investigators (MI and KS) then classified the clinically related educational and non-educational videos. Subsequently, the principle (HMA) investigator (NK), an expert in “content analysis,” reviewed all the educationally useful videos. A standard data-collection form was used to ensure the accuracy of the collected data by strictly following the pre-set inclusion/exclusion criteria. The major characteristics summarized from the retrieved videos included, the title of the video, total length of the video (in min/s), total no of the views/viewers of the video, total number of likes or dislikes, date of upload, positive and negative comments associated with the video, and name of the publisher or uploader of the video. The matters relating to disagreement on any item of the data from retrieved videos were fully debated with the investigators/subject experts/content analyst in the presence of an adjudicator (SH) to achieve a final consensus.

### 2.3 Inclusion and exclusion criteria for YouTube videos

**Figure 1:**
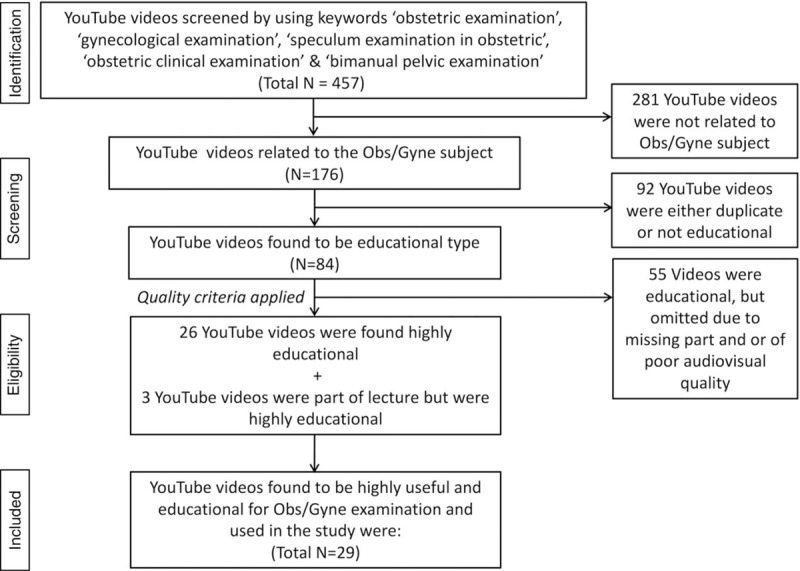
PRISMA 2009 flow-diagram: showing the selection procedure following the pre-set inclusion/exclusion criteria of Obs/Gyne physical examination videos available on YouTube. Obs/Gyne = obstetrics and gynecology, PRISMA = Preferred Reporting Items for Systematic Reviews and Meta-Analyses.

All the published YouTube videos included in the present study had to meet the given basic criteria for educationally useful video, that is, the video must provide the scientifically correct knowledge along with complete clinical and physical instruction skills. The YouTube videos must have been published in the English language. YouTube Obs/Gyne videos with same content (with same or varying time length), published by more than one publisher/source or videos with more than one title were considered duplicate and disqualified straightaway. In addition to the above, when the same video/content appeared in several YouTube videos (published with different names), only the most recent one or the complete video was included in this study. One of the major reasons for internet video exclusion from this study was duplication of the video. On the contrary, the criteria for the designation as “non-educational videos” were: “not well presented,” “inferior visualization,” misleading knowledge, scientifically unproven information, not in the English language, “in the form of a lecture” only, an advertisement or news, documentary or movie clips. Furthermore, the date of posting, numbers of views/viewers, number of likes and dislikes, and the maximum number of positive comments were summarized. Additionally, a manual search of the “suggested videos” list from the retrieved YouTube videos result pages was also performed for additional, eligible pertinent videos. The selection process of YouTube videos of Obs/Gyne physical examination employing pre-set inclusion/exclusion criteria is given in PRISMA 2009 flow-diagram (Fig. 1).

### 2.4 Statistical analysis

The data collected from the reviewed YouTube videos were summarized using a standard form and entered into Microsoft Excel (MS Excel) version 2013 and analyzed using SPSS version 22.0 statistical software package (IBM). Descriptive statistics (mean, standard deviation [SD], etc) were used for analyzing the data and outcome variables. Statistical *t* test (analysis of variance [ANOVA]) was performed to determine the *t* value and significant differences. Pearson correlation coefficient (*r*) was employed to establish a correlation between like versus dislike, like versus total views/d, like versus like/d, and like versus dislike/d for all the “Educational videos” under investigations. The statistical significance level *P*-value <0.05 was maintained during the entire analysis. The videos’ credibility was measured as varied scores ([Like–Dislike]/[Like, Dislike] × 100) and the videos’ merits ([Positive comments–Negative comments]/[Total comments] × 100).

### 2.5 Ethical approval

This study is exempted from ethical approval as it is a type of systematic review performed using internet based search and there is no involvement of patient/healthy subjects during the entire course of study.

## 3 Results

After numerous rounds of screening and review of all the 457 retrieved YouTube videos, only 84 (18.3%) videos were found relevant and educational according to the set criteria. A total of 84 videos were found to have information of Obs/Gyne physical examinations. After reviewing these 84 videos based upon our pre-set inclusion/exclusion criteria, only 29 (34.52%) videos were found to be educationally useful and the remaining 55 (65.47%) videos were misleading or useless from an educational point of view. The mean duration of these videos was 397 seconds (SD = 302.77) (Table 1).

**Table 1:**
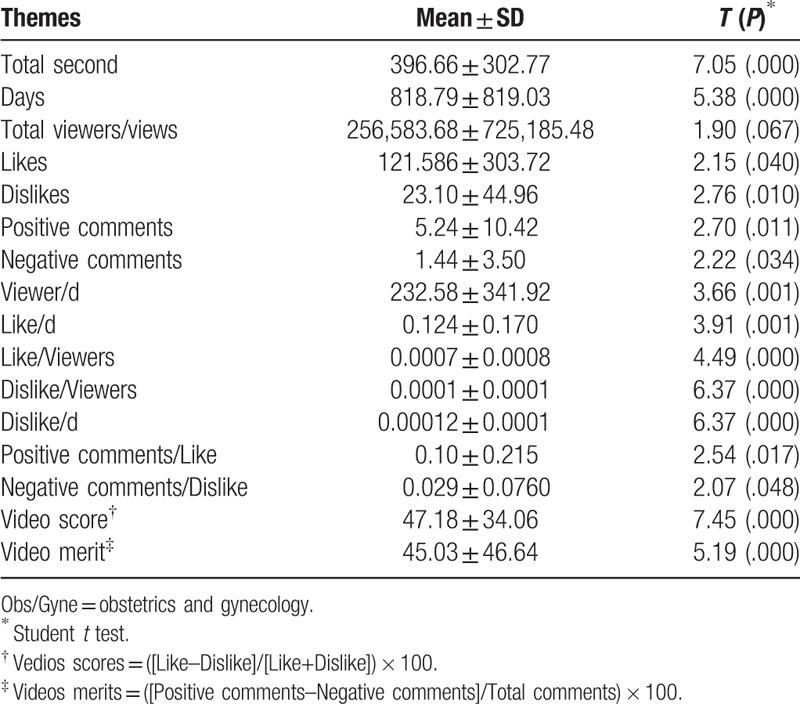
Needful information and mean scores of all the 29 YouTube Obs/Gyne videos included in this study.

The duration of educationally useful videos was 21 to 6196 seconds (Table 2). The mean viewership or views/d of the videos included in the study was 232.58 (SD = 341.92) and the range of viewership of the educationally useful videos was 0.8 to 1307.3 views/d. A total mean of “likes” for all of the 29 educationally useful videos was 121.5 (SD = 303.7), which showed that a significant number of users like the educationally useful Obs/Gyne videos (*t* = 2.15, *P* = .04). The average number of “liked” videos by the viewers/d, when the users visit the YouTube, the mean of those videos was 0.12. Due to login information requirement in order to “like,” “dislike,” or comment on any YouTube video, most of the users just view the video rather like, dislike and/or comment on it. The total mean of “dislikes” of educationally useful videos was 23.10 (SD = 44.96) which also showed that some difference of opinions were present (*t* = 2.76 and *P* = .01). The minimum number of positive comments left for each educationally useful video was 0 and the maximum was 50, with a mean of 5.24 (SD = 10.42). The total mean of negative comments was 1.44 (SD = 3.50).

**Table 2:**
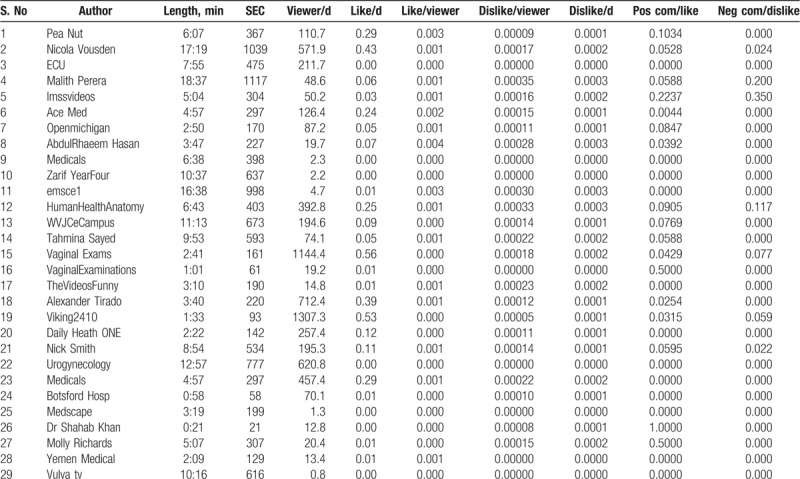
Detailed information of all the 29 videos included in the study.

The detailed information about the 29 educationally useful videos included in the present study for the review purpose has been shown in Tables 2 and 3. It is important to note that all the 29 educationally useful videos were created and published by professional doctors, expert bodies, or university teaching clinics. Additionally, the links for the concerned publishing organizations, publishers, or authors were provided and included AceMed, PeaNut, ECU, UCUrogynecology. The range of all the 29 videos inter-rater reliability was between 0.50 and 0.96.

**Table 3:**
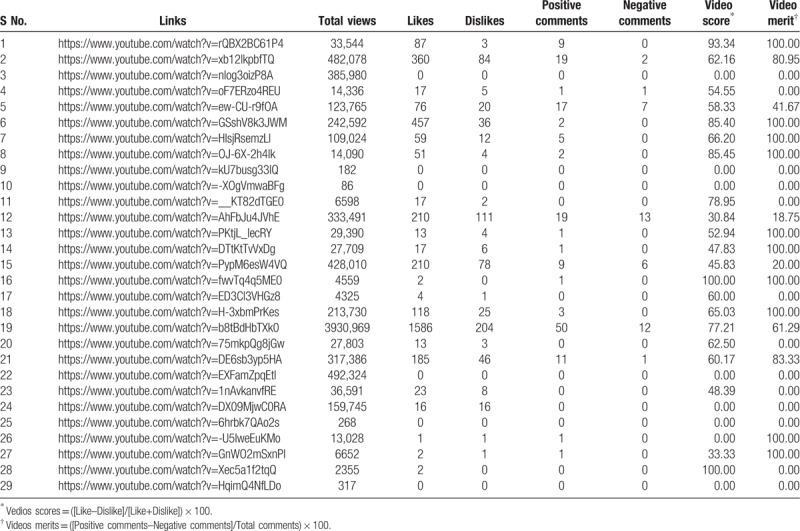
Access links and related comments of all the 29 videos included in the study.

The correlation between the total 29 educationally useful videos views and the number of viewers/d were high and showed positive correlation with the result being statically significant (*r* = 0.72, *P* = .000). Likewise, significant correlation was found between the total views and the like/d (*r* = 0.54, *P* = .002), but no significant correlation was found with video scores (*r* = 0.13, *P* = .48) and video merit (*r* = 0.64, *P* = .74) (Table 4).

**Table 4:**
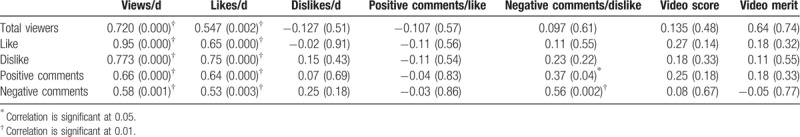
Correlation between the likes/dislikes, comments, video score, and merit of 29 videos.

The total likes of all 29 videos and the number of views/d showed statistically significant correlation (*r* = 0.95, *P* = .000). However, no correlation was found between the video scores (*r* = 0.27, *P* = .14) and video merits (*r* = 0.18, *P* = .32). A significant correlation was also found between the total dislikes and views/d, likes/d, while the relationship with the score of the video and the merits of the video were not significant (Table 4).

## 4 Discussion

To the best of our knowledge, this is the very first study involving a systematic review of YouTube videos of Obs/Gyne physical and clinical examinations for assessing the educational usefulness and accuracy of these internet videos. In this study we found that a significant number of viewers used YouTube videos as a learning resource for the Obs/Gyne physical examination. Most of the videos available on YouTube related to the Obs/Gyne physical examination were available from individuals with unknown credentials, while only limited videos were available from the professional sources. Recent studies have shown that the diversity of the publishers/authors and complete lack of peer-review process on the YouTube platform have led to the posting/publishing of inappropriate videos containing inaccurate or misleading health information.^[1,2]^ Interestingly, this study also observed that YouTube search engine displays a large number of pages, however, only a small number of initial web-pages were useful and relevant to the “search keyword.” Several studies have revealed that a large number of web-pages, mostly display either repetitive videos, very short and inaccurate videos, or show information related to advertisements, marketing, and promotions.^[10,15]^ Obs/Gyne physical examination videos published from some authentic sources showed more likes by viewers rather than other sources, possibly because the authentic sources are considered as more reliable resources. We also noticed that educationally useful videos that linked with clinics, hospitals, universities, or any other educational organizations are contributing more towards the progress of knowledge and skills in terms of a learner's resource. These findings are in line with a previously published study of Azer et al.^[15]^ The statistical analysis of the data of this study revealed significant growth of educationally useful video users, that is, those who visited and/or liked the Obs/Gyne physical examination videos. Only a very small percentage of the users dislike the videos tagged as educationally useful. Most of the YouTube videos of Obs/Gyne physical examination, which were useful and accurate, showed positive correlation with the total likes versus total number of views/d, and total likes versus likes/d.

The benefits and success of video-based self-instruction learning have previously been demonstrated in several studies.^[16–19]^ It has already been reported that the educational videos are an efficient medium of learning that can assist in mastering skills through repeated watching of the video showing the used techniques and following the given information.^[15]^ In addition, the learners attending the clinical skills sessions along with watching the videos (online or off line) and practicing the learned skills on the patients have become one of the most important learning strategies in most medical schools.^[20,21]^ This study also demonstrated that YouTube videos, which were judged for accuracy, scores on the basis of likes and dislikes; were almost >50%. Overall, the current findings suggests that some of the Obs/Gyne physical examination videos available on YouTube are potentially useful self-learning resources for active learners and help in gaining knowledge and skills, as shown the majority of the educationally useful videos having high like scores. YouTube videos that are academic and professional in nature, and when used properly, reinforce students’ learning, possibly due to audio/video clarity and the content of the video.^[22]^ An earlier study reported that a good number of the students utilize their self-study time to learn skills by the use of internet videos and scientific articles.^[23]^

The comments posted against each educationally useful YouTube video of Obs/Gyne physical examination were reviewed during the data collection process, and it was found that most of the viewers were healthcare professionals, trainees, and apprentices. It was noticed that many users view the videos but they did not like, dislike, or post comments, possibly due to login information required for all the above mentioned activities. The merit score of Obs/Gyne physical examination videos on the basis of positive and negative comments was >40%. Most of the negative comments were highlighting the need of betterment of YouTube's search algorithm system for finding an easy way to the related keywords search item and generating more precise lists of response (i.e., videos).^[24–26]^ Positive comments from viewers related to an appreciation of the Obs/Gyne physical examination videos of YouTube as educationally knowledgeable, authentic, and accurate, along with good presentation.^[26]^

In view of increasing utilization of YouTube videos as a learning resource in our day-to-day learning, our current findings support the on-going effort of discussing, evaluating, and improving the Obs/Gyne physical examination videos available on the YouTube platform.^[14]^ The quality of these educational video clips can easily be improved, when healthcare organizations and academic institutions realize their impact and act upon the need for producing high quality medical multimedia resources for healthcare professionals and medical students.^[14]^ YouTube videos can be used for training and education purposes, but the selection of the videos should be done with utmost care considering underlying medical standards, completeness of information provided, and procedural correctness. Earlier studies have shown the effectiveness of video-based self-instruction learning without the assistance of a mannequin.^[27–29]^ Keeping the current trend of learning, globalization, and intervention of information technology in society, internet videos (especially YouTube videos or any other video sharing website or videos available through internet based online channels) are one of the best ways of learning for all age groups. As, the popularity of YouTube is significant, the quality of YouTube videos, especially those dedicated to educational and training purposes, must be very high. Our study provides a contemporary evaluation of YouTube videos of Obs/Gyne physical examination in terms of their content and user engagement and gives insight for improving the quality of videos for their active usage for learning purposes in clinical training.

### 4.1 Limitations of the study

The assessment of the Obs/Gyne physical examination videos available on YouTube was performed for available and accessible videos during the period between Novamber 11, 2015 and March 15, 2017. Since then, many more videos might have been uploaded and some relevant information and content might have changed over time. As, we excluded non-English language videos in this study, the chances are there that pertinent good quality Obs/Gyne physical examination videos might have been published in other languages that may have affected the outcome of this study. In addition, at different time points, the search results may vary. In general, YouTube performs search according to the default setting of “relevance” but search rankings may vary temporarily by geographic location, or by other undisclosed factors. In this study, we performed YouTube searches according to the default setting of “relevance.”

## 5 Conclusions

In conclusion, Obs/Gyne physical examination multimedia videos uploaded by healthcare professionals or institutes on YouTube were found to be a good source of information for medical education and healthcare service training. Peer reviewed videos of educational usefulness with known publisher identity can be used by medical students/practitioners for self-instruction learning and by clinical teachers as learning resources. We summarize that YouTube is a dominant free online platform for the posting of videos, and clinicians or other healthcare workers, along with the consumers, must be aware of the source and evaluate the intent and accuracy of the content of the video before accepting/using it for training and educational purposes.

## Acknowledgments

The authors are grateful to Dr Shaun Cochrane, a native English speaker, currently holding a position of Deputy Research Manager, Guys and St Thomas Hospital, London, UK, for proofreading the manuscript. Also, the authors sincerely acknowledge the software program related service support for the statistical analysis provided by the College of Medicine, King Saud University, Riyadh, Saudi Arabia. The author, Dr Shafiul Haque is grateful to the Deanship of Scientific Research, Jazan University for providing the access of Saudi Digital Library for this study.

## Author contributions

**Conceptualization:** Hamza Abdulghani, Shafiul Haque, Tauseef Ahmad, Mohammad Irshad, Kamran Sattar, Mohammed Meteb Al-harbi, Nehal Khamis.

**Data curation:** Hamza Abdulghani, Shafiul Haque, Tauseef Ahmad, Mohammad Irshad, Kamran Sattar.

**Formal analysis:** Hamza Abdulghani, Shafiul Haque, Tauseef Ahmad, Mohammad Irshad, Kamran Sattar, Mohammed Meteb Al-harbi.

**Funding acquisition:** Hamza Abdulghani, Mohammed Meteb Al-harbi.

**Investigation:** Hamza Abdulghani, Shafiul Haque, Tauseef Ahmad, Mohammad Irshad, Kamran Sattar.

**Methodology:** Hamza Abdulghani, Shafiul Haque, Tauseef Ahmad, Mohammad Irshad, Kamran Sattar, Mohammed Meteb Al-harbi, Nehal Khamis.

**Project administration:** Hamza Abdulghani, Mohammed Meteb Al-harbi, Nehal Khamis.

**Resources:** Hamza Abdulghani, Kamran Sattar, Mohammed Meteb Al-harbi, Nehal Khamis.

**Software:** Tauseef Ahmad, Mohammed Meteb Al-harbi.

**Supervision:** Hamza Abdulghani, Shafiul Haque, Nehal Khamis.

**Validation:** Hamza Abdulghani, Tauseef Ahmad, Mohammad Irshad, Kamran Sattar, Mohammed Meteb Al-harbi.

**Visualization:** Hamza Abdulghani, Tauseef Ahmad, Mohammad Irshad, Kamran Sattar, Nehal Khamis.

**Writing – original draft:** Hamza Abdulghani, Shafiul Haque, Tauseef Ahmad, Mohammad Irshad.

**Writing – review & editing:** Hamza Abdulghani, Shafiul Haque, Nehal Khamis.
